# Comparative Transcriptome Analysis of *Babesia bigemina* Attenuated Vaccine and Virulent Strains of Mexican Origin

**DOI:** 10.3390/vaccines12030309

**Published:** 2024-03-15

**Authors:** Rebeca M. Santamaria, Karel Estrada, María E. López, Edith Rojas, Grecia Martínez, Yazmín Alcalá, Carmen Rojas, Jesús Antonio Álvarez, José J. Lira, Tomás V. Santamaria, Alejandro Sánchez-Flores, Julio V. Figueroa

**Affiliations:** 1 Centro Nacional de Investigación Disciplinaria en Salud Animal e Inocuidad, Instituto Nacional de Investigaciones Forestales, Agrícolas y Pecuarias (INIFAP), Jiutepec 62550, Morelos, Mexico; santamaria.rebeca@inifap.gob.mx (R.M.S.); mlopez.arellano@gmail.com (M.E.L.); martinez.grecia@inifap.gob.mx (G.M.); rojas.carmen@inifap.gob.mx (C.R.); alvarez.jesus@inifap.gob.mx (J.A.Á.); lira.juan@inifap.gob.mx (J.J.L.); valdemarsma@gmail.com (T.V.S.); 2 Facultad de Medicina Veterinaria y Zootecnia, UNAM, Mexico City 04510, Ciudad de México, Mexico; yazmin@unam.mx; 3 Instituto de Biotecnología, Unidad Universitaria de Secuenciación Masiva y Bioinformática, UNAM, Cuernavaca 62209, Morelos, Mexico; karel.estrada@ibt.unam.mx (K.E.); alejandro.sanchez@ibt.unam.mx (A.S.-F.); 4 Centro Nacional de Recursos Genéticos, INIFAP, Tepatitlán de Morelos 47600, Jalisco, Mexico; rojas.edith@inifap.gob.mx

**Keywords:** *Babesia bigemina*, transcriptome profiling, differentially expressed genes

## Abstract

Bovine babesiosis, caused by the protozoan *Babesia bigemina*, is one of the most important hemoparasite diseases of cattle in Mexico and the world. An attenuated *B. bigemina* strain maintained under in vitro culture conditions has been used as a live attenuated vaccine; however, the biological mechanisms involved in attenuation are unknown. The objective of this study was to identify, through a comparative transcriptomics approach, the components of the *B. bigemina* virulent parasites that are differentially expressed in vivo, as opposed to those expressed by *B. bigemina* attenuated vaccine parasites when inoculated into naïve cattle. The biological material under study was obtained by inoculating spleen-intact cattle with infected erythrocytes containing either the attenuated strain or a virulent field strain. After RNA extraction, transcriptomic analysis (RNA-seq) was performed, followed by bioinformatic Differential Expression (DE) analysis and Gene Ontology (GO) term enrichment. The high-throughput sequencing results obtained by analyzing three biological replicates for each parasite strain ranged from 9,504,000 to 9,656,000, and 13,400,000 to 15,750,000 reads for the *B. bigemina* attenuated and virulent strains, respectively. At least 519 differentially expressed genes were identified in the analyzed strains. In addition, GO analysis revealed both similarities and differences across the three categories: cellular components, biological processes, and molecular functions. The attenuated strain of *B. bigemina* derived from in vitro culture presents global transcriptomic changes when compared to the virulent strain. Moreover, the obtained data provide insights into the potential molecular mechanisms associated with the attenuation or pathogenicity of each analyzed strain, offering molecular markers that might be associated with virulence or potential vaccine candidates.

## 1. Introduction

*Babesia bigemina* is an intraerythrocytic protozoan of the phylum Apicomplexa and is one of the main parasite species of bovine babesiosis in Mexico and the world. The dissemination of this disease is constrained to tropical and subtropical regions, and it is considered one of the foremost vital diseases vectored by *Rhipicephalus microplus* and *Rhipicephalus annulatus* ticks [1]. In Mexico, the tropical and subtropical regions represent approximately 53% of the national territory, and more than 75% of cattle farming is found in these regions [2]. This means that from the national record of approximately 33.5 million cattle, 25.1 million are found in areas of high endemicity. In 2016, an economic estimation for annual losses was ~68,878,694 USD, showing decreased milk production due to the presence of *R. microplus*, whereas in beef cattle (*Bos indicus* × *Bos taurus*) in Mexico, a loss of USD 504,729,382 was only estimated by the vector [3]. However, today, there is a worldwide concern about economic losses generated by bovine babesiosis and problems with animal health. As control and prevention strategies, different methods have been used for decades; for instance, tick control with ixodicides, development of genetically resistant cattle [4,5], and inducing an active immune response through available attenuated live vaccines against *B. bigemina*, resulting in high protection [6,7,8]. While it has been shown that there are antigenic similarities and differences between *Babesia bigemina* and *B. bovis* [9,10], it is known that a satisfactory cross-immunity between the two species does not happen [11,12]. Hence, immunization would be the method that provides the best prospects of preventing and controlling bovine babesiosis [6,7,8,13]. In addition, several studies have been published to identify potential vaccine material, some of which are based on lysates of *Babesia*-infected erythrocytes or *Babesia* sp. culture supernatants, including *Babesia bovis* and *B. bigemina* [7,8,14,15]. The use of recombinant subunits and synthetic peptide-based vaccines has also been recently reviewed [16,17]. In Australia, the use of live attenuated vaccines obtained by multiple passages in splenectomized calves has been well described [6,7,8,16,17]. An experimental live attenuated vaccine has been developed and tested in vaccinated cattle under a variety of controlled needle-challenge and tick-transmitted *Babesia* natural challenges, showing high efficacy results. The studies carried out by our research group allowed us to show that the *B. bigemina* strain maintained by continuous in vitro culture behaved as an attenuated and safe parasite population, because that vaccine did not seriously affect hematological values when inoculated into susceptible animals [8,12,13,18,19,20]. An attenuated strain of *B. bigemina* was tested, inducing protection against heterologous challenge with infected blood or infected ticks in the field. The vaccine strain provides adequate protection without tick infection, which is considered ecologically desirable because it prevents the occurrence of clinical disease caused by the spread of the vaccine strain [8,18].

It has been hypothesized that the absence of selective pressure during the prolonged maintenance of a *B. bigemina* strain in continuous in vitro culture has induced structural changes in its genome, as evidenced through comparative genomics with a Mexican wild type *B. bigemina* virulent population [21]. However, little is known about the most relevant genes differentially expressed from *B. bigemina* in vitro culture used as live attenuated *vaccine*. The aim of this study is to identify, through a comparative transcriptomics approach, the components of the *B. bigemina* virulent parasites that are differentially expressed in vivo, as opposed to those expressed by *B. bigemina* attenuated vaccine parasites when inoculated into naïve cattle. A bioinformatics analysis of the differential expression of genes in the two groups of cattle will facilitate a broader understanding of the biology of the virulent strain, particularly with regard to the virulent factors involved in the pathogenicity towards the bovine host.

## 2. Materials and Methods

### 2.1. Sample Collection

#### 2.1.1. *Babesia bigemina* Virulent Strain

The field isolate of *B. bigemina* was originally obtained from a clinical case in Mexico [12]. It was kept as a stabilate in cryopreservation and maintained by alternate passages in ticks and splenectomized cattle only to reactivate the virulent strain when needed [13,21].

#### 2.1.2. *B. bigemina* Attenuated Strain

The attenuated strain of *B. bigemina* is a population of parasites originally derived from the same virulent isolate collected in Mexico during a clinical case of babesiosis. The strain was adapted to in vitro culture using a stationary microaerophilic system [19], and, since then, it has been maintained alternately in continuous culture and cryopreservation [20,22,23].

#### 2.1.3. Experimental Cattle

**Figure 1:**
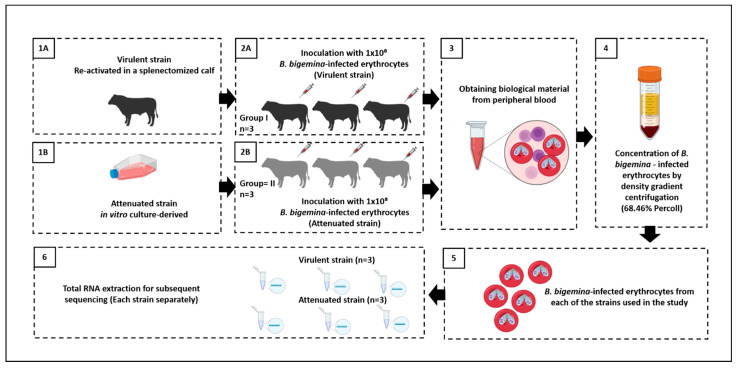
Illustration of the experimental design to obtain biological material for the research study. (**1A**) Activation of the *B. bigemina* virulent strain in a splenectomized calf. (**1B**) In vitro culture to obtain the *B. bigemina* attenuated (vaccine) strain. (**2A**,**2B**) inoculation of steers by deep intramuscular route, with the specified dose for each of the *B. bigemina* strains (n = 3, for both groups). (**3**) Obtaining the biological material from each of the inoculated steers. (**4**) Infected erythrocyte concentration performed with a 68.46% Percoll density gradient. (**5**) Concentration of infected erythrocytes. (**6**) Extraction of total RNA from each of the samples obtained (3 biological replicates for each *B. bigemina* strain).

A total of seven *Bos taurus* steers free of bovine brucellosis and tuberculosis, as required by the animal diseases zoosanitary campaigns at the national level, but also free of *Rhipicephalus microplus*, and, therefore, free of bovine babesiosis, were purchased from a beef cattle farm located in the high plateau of central Mexico. Selected animals were confirmed negative for babesiosis by using serological and molecular diagnosis methods such as the Indirect Fluorescent Antibody Test (IFAT) and nested-PCR assay, respectively [20,22]. One of the steers was splenectomized to reactivate the virulent strain, which had been kept cryopreserved in liquid nitrogen. After the recovery of the virulent strain, three steers were inoculated with an infected-erythrocyte dose of 1 × 10^8^ by the IM route (Group I). The in vitro culture-derived attenuated strain was inoculated into three steers with a similar dose of 1 × 10^8^ by the IM route (Group II) (Figure 1). The handling of the experimental animals during the study was carried out following good management practices for animal welfare at CENID-SAI, and with the approval provided by the Institutional Subcommittee for the Care and Use of Experimental Animals (SICUAE.DC-2022/1-3) at FMVZ-UNAM.

#### 2.1.4. Clinical Monitoring

The *B. bigemina*-inoculated splenectomized calf as well as the Group I steers were subject to daily clinical monitoring, recording the rectal temperature value and clinical signs associated with *B. bigemina* infection such as hemoglobinuria [22,23]. Blood sampling by coccygeal vein puncture was obtained to determine the Packed Cell Volume (PCV) using the microhematocrit technique. This parameter is essential in monitoring a case of clinical babesiosis, since it reveals the percentage of the volume of erythrocytes in the peripheral blood. When the percentages are low (<25%), and mainly associated with the presence of *Babesia bigemina*, it is indicative of the clinical severity of the disease due to hemolysis of the erythrocytes. Giemsa-stained blood smears for microscopic examination were implemented to assess the percentage of parasitized erythrocytes (PPE). A PPE ≥3%, fever ≥40 °C, a decrease in microhematocrit ≥20–30% of the baseline value, the presence of hemoglobinuria, anorexia, and animal prostration were parameters indicative that the strain was virulent. After obtaining the virulent parasite population at peak parasitemia, steers were treated with diazoaminodibenzamidine diaceturate and supportive therapy. The steers inoculated with the attenuated vaccinal strain were also clinically monitored as described above; however, as they did not show clinical manifestations of disease, no treatment was initiated, while the steers inoculated with the virulent strain were treated to resolve the clinical disease.

#### 2.1.5. Concentration of *Babesia bigemina*-Infected Erythrocytes

An iso-osmotic gradient with 68.46% Percoll was used essentially as described before [23]; specifically, by mixing 30 mL of Percoll (SIGMA Chemical) and 4.8 mL of blood from the inoculated steers in VYM solution (V/V) in a transparent polycarbonate tube of 14 × 70 mm, and centrifuging it for 6 min at 30,000× *g* at 4° C, with deceleration. Infected erythrocyte-containing fractions were collected to subsequently perform three washes with VYM at 4000 r. p. m. for 15 min at 4 °C [24]. Finally, the fractions were separated, and Giemsa-stained smears were made. The pellet containing the concentrated infected erythrocytes was later frozen at −80 °C with RNA for the subsequent extraction of the total RNA.

### 2.2. RNA-Seq

#### Total RNA Extraction

**Figure 2:**
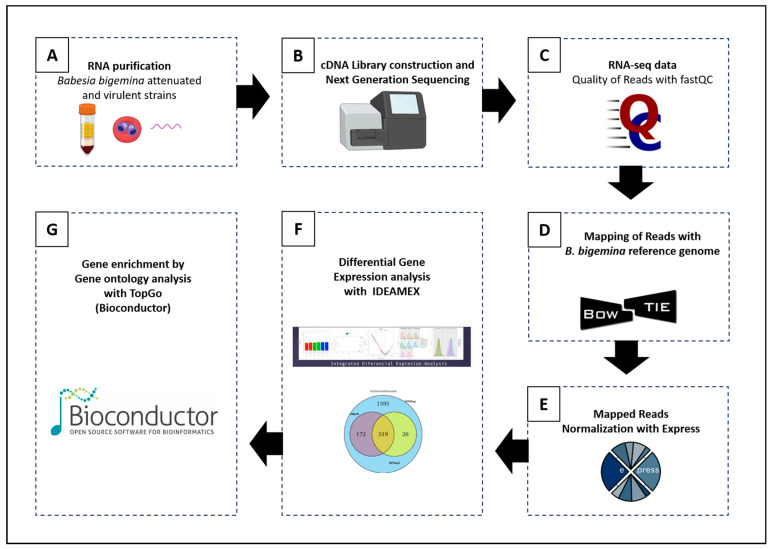
Workflow for the RNA-seq analysis of *B. bigemina* attenuated (vaccine) and virulent strains. (**A**) Sample preparation (total RNA). (**B**) Sequencing with the Illumina MiSeq Next Generation Sequencing Technology platform (Sanger/Illumina 1.9), obtaining the sequence “reads” in fastq formats. (**C**) Primary analysis using the FastQC program (version 0.11.5). (**D**) Cleanup of sequence reads by filtering out reads that mapped to the *Bos taurus* genome (GCF_002263795.1), and alignment of the filtered reads by mapping to the *B. bigemina* reference genome (GCF_000981445.1). (**E**) Quantitation and normalization of data. (**F**) Differential expression analysis with the IDEAMEX platform, using the edgeR, DESeq2 and NOISeq programs. (**G**) Functional annotation performed by assigning the gene ontology (GO) categories, with the R program “TopGO” (v2.46.0). (Partial images supported by BioRender.com) (accessed on 2 November 2023).

The workflow applied in this study, including the experimental design and the bioinformatics process, is shown in Figure 2. The total RNA of the *B. bigemina* parasites was extracted using the Rneasy Mini commercial kit (Qiagen^®^, Hilden, Germany) with slight modifications (Optional use of DNAse). The obtained RNA molecules were evaluated for integrity by agarose gel electrophoresis to determine quality and abundance [25]. Likewise, the RNA samples were quantified using an Implen NanoPhotometer^®^ (Implen, Munich, Germany). Finally, the RIN (RNA Integrity Number) was determined utilizing a profile generated by the Agilent 2100 equipment (Agilent, Santa Clara, CA, USA) [26,27].

### 2.3. Sequencing

The Illumina platform, MiSeq Next Generation Sequencing Technology (Sanger/Illumina 1.9) with a phred33, was used to sequence the transcripts for both *B. bigemina* strains. The transcriptomes were generated in the University Unit of Massive Sequencing and Bioinformatics of the Institute of Biotechnology-UNAM, using a minimum of 3 µg of total RNA of the highest possible quality to construct paired and strand-specific read libraries with the TruSeq RNA Library Preparation Kit (Illumina, San Diego CA, USA).

### 2.4. Bioinformatic Analysis

The quality control of the reads was conducted using FastQC v0.11.5 [28]. The high-quality reads were then mapped to the reference genomes of *B. bigemina* (GCF_000981445.1) and *B. taurus* (GCF_002263795.1) using the bwa v0.7.17-r1188 software. Subsequent filtering steps were applied to separate the reads that mapped using Samtools v1.13 and Bamtools v2.3.0 suites. Counting and normalization of the mapped reads were carried out using the Express program (v1.5.1). Finally, the differential expression analysis was performed using the three biological replicates for each of the *B. bigemina* strains compared in this study; the IDEAMEX web server tool “IDEAmex—Integrated Differential Expression Analysis MultiExperiment (unam.mx)” (accessed on 31 July 2023) was used for this [29]. Three differential expression software packages, namely, edgeR [30], DESeq2 [31] and NOISeq, [32] were applied. To report differentially expressed genes, a cut-off line of <0.01 for “False Discovery Rate” (FDR) and a >2 logarithm of the fold change (logFC) were set [32].

### 2.5. In Silico Homology Analysis of Genes with Other Apicomplexa Organisms

The upregulated genes in *B. bigemina* were translated into proteins and used as queries to identify potentially orthologous genes in *Apicomplexa* organisms, particularly within the genus *Babesia*. This process was executed using the BLAST aligner software (version 2.2.18) [33] against the non-redundant (nr) protein database from NCBI, BLASTp (protein/protein BLAST, part of the suite of Standard Protein BLAST at NCBI (https://blast.ncbi.nlm.nih.gov/Blast.cgi?PAGE=Proteins) (accessed on 22 September 2023), and all results underwent manual inspection.

### 2.6. Gene Ontology Enrichment

Using a GO term enrichment analysis of differentially expressed genes, different protein families were identified. This was accomplished using the R program “TopGO” (v2.46.0). GO terms with a *p*-value < 0.05 were considered significantly enriched (Figure 2) [34].

## 3. Results

### 3.1. Obtaining Biological Material

#### 3.1.1. Splenectomized Calf

The re-activation of the virulent strain was successfully achieved by splenectomizing and later inoculating the calf, obtaining the following results throughout the clinical monitoring: on day 5 post-infection (PI), clinical signs associated with bovine babesiosis were reported as fever 41 °C, hemoglobinuria, PCV value 16% and PPE ≥ 10%. Based on this information, it was decided to obtain biological material to inoculate three corresponding bulls of Group I with a dose of 1 × 10^8^ infected erythrocytes (IE). After bleeding, the calf was treated with diasaminodibenzamide diaceturate in addition to supportive care.

#### 3.1.2. Virulent Strain

The three steers inoculated with the virulent dose (recovered from the splenectomized calf) began to present fever (>40 °C) on day 4 PI, and the PCV value decreased from day 5 PI onwards, reaching 11.3% in one of the steers at the end of the monitoring (Supplementary Figures S1 and S2). The presence of the protozoan parasite was determined by light microscopy by examining blood samples on days 5–9 PI. The biological material on one steer was taken at peak parasitemia on day 8 PI, and on day 9 PI on two other steers (estimated PPEs ≥ 8%). The steers were later treated with the same agents as the splenectomized calf to prevent death.

#### 3.1.3. Attenuated Strain

Steers vaccinated with the attenuated strain (G II) did not develop fever during the observation period (Supplementary Figure S1). Regarding the PCV, there was only a slight decrease and moderate clinical signs on PI days 3–7 (Supplementary Figure S2), indicative of animals infected with live attenuated parasites [7,12]. It is important to mention that none of the bulls in Group II required treatment against babesiosis; the PCV returned to baseline values on day 9 PI and the PPE assessed was minimal (≤0.1%) on days 6–9 PI. All three GII steers were bled on day 8 PI. This is particularly important since the low percentage of parasite erythrocytes reached was due to the strain’s intrinsic lack of virulence.

#### 3.1.4. Concentration of *Babesia bigemina*-Infected Erythrocytes

To obtain the concentrated *B. bigemina*-infected erythrocytes, a 68.46% Percoll density gradient protocol was used, which, after high-speed centrifugation, allowed for the recovery of three fractions: the upper thin fraction, an intermediate fraction, and a lower fraction. Microscopic analysis of these three fractions indicated that the thin upper phase contained mainly red cell membranes and some erythrocytes infected with *B. bigemina* trophozoites, while the middle phase contained most of the erythrocytes infected with *B. bigemina* trophozoites and merozoites. Finally, in the lower phase of the gradient, there were mostly bovine mononuclear cells (Supplementary Figure S3).

### 3.2. Total RNA Extraction

A total of six RNA extractions were performed, three corresponding to *B. bigemina*-erythrocytes obtained from steers inoculated with the virulent strain (G I) and three steers inoculated with the attenuated strain (G II), complying with the RNA concentrations and quality parameters required for sequencing. The total RNA samples were found to be free of phenolic contaminants and proteins when quantified with the Nanodrop equipment. Likewise, when analyzed with the Bioanalyzer 2100, RIN numbers were considered adequate for RNA-seq.

### 3.3. Sequencing

A total of six cDNA paired-end libraries, representing three biological replicates for each of the *B. bigemina* strains analyzed in this study, were built. The sequencing analysis with the Sanger/Illumina 1.9 platform-generated range extends from the lowest to the highest values across different replicates of the same strains, obtaining 9,504,000–9,656,000 and 13,400,000–15,750,000 sequence reads for the attenuated strain replicates and the virulent strain replicates, respectively, with a sequence length of 76 bp and 53% of GC (Table 1). The analysis with the FastQC software (Version 0.12.0)revealed good sequence quality, with a Phred value of >25. 

### 3.4. Bioinformatic Analysis

**Figure 3:**
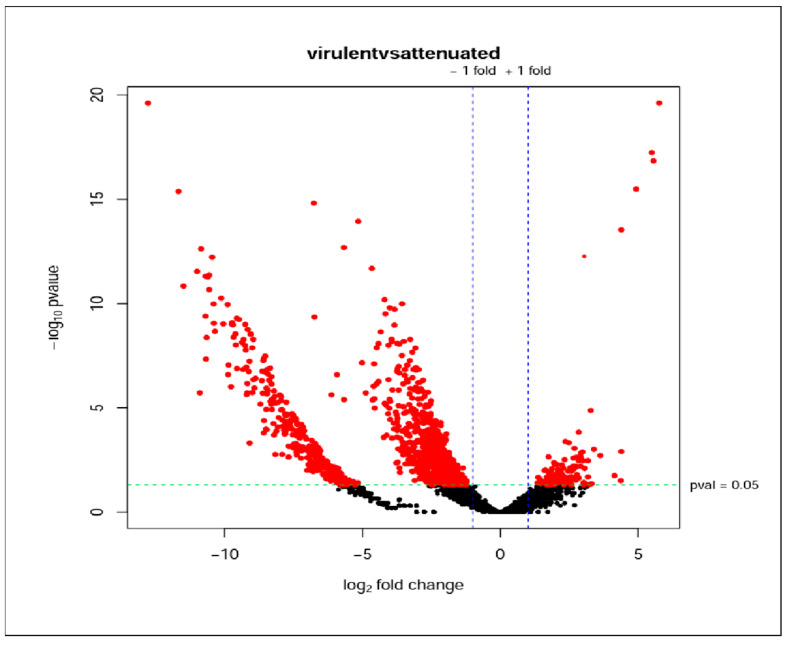
Volcano plot of differentially expressed genes (DEGs) between virulent and attenuated strains of *Babesia bigemina*. The *X*-axis is the logarithmic scale 2 of the fold change in gene expression (log2 (fold change)). The *Y*-axis is the log10-adjusted *p*-value, indicating the level of significant difference in expression. Black dots represent genes without differential expression; red dots represent differentially increased genes based on p-adj and logFC cut-off values.

The RNA-seq reads were mapped against the reference genome of the *B. bigemina* BOND strain. Concerning the similarity and variation between the biological replicates, the multidimensional scaling (MDS) method reveals a significant similarity, particularly among the triplicates belonging to the virulent strain (Supplementary Figure S4). Additionally, a slightly larger count per million (CPM) was obtained in these than in the attenuated strains, as shown in Supplementary Figure S5. The global assembly of the *Babesia bigemina* mapped transcripts was used for the differentially expressed gene (DEG) analysis. The DEGs were identified with the criteria of log2 (fold change) > 2 and an adjusted *p*-value < 0.01 for comparing differential expression patterns between the virulent strain and the attenuated strain of *B. bigemina*. A total of 3544 genes were detected, with 691 identified using the edgeR software (version 4.0.16), 2308 with NOIseq (version 1.42.1), and 545 with DESeq2 (version 1.42.1). The results of the total differentially expressed genes between the two *Babesia bigemina* strains can be visualized in the volcano plot, represented with red dots (Figure 3).

**Figure 4:**
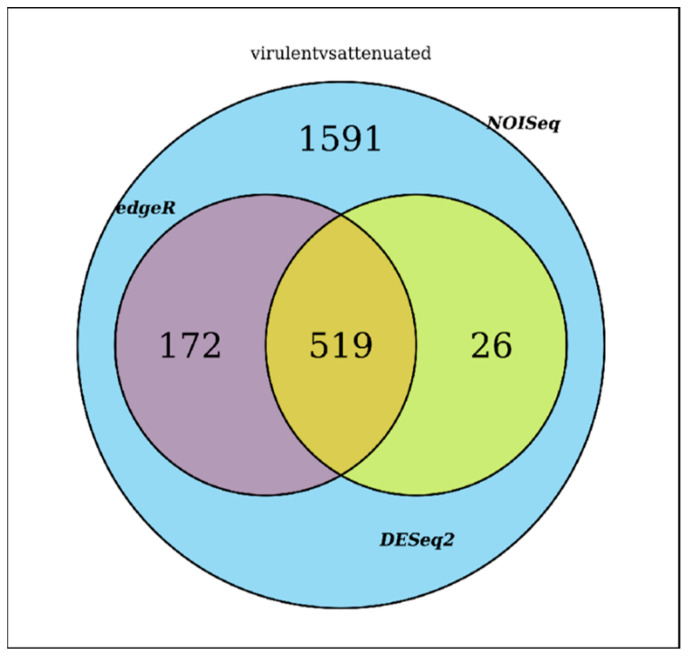
Venn diagram. Integration summary with the intersection for DE genes identified with NOISeq, edgeR, and DESeq2.

For our study, we focused on the intersection of the three methods as the most reliable, resulting in a total of 519 differentially expressed genes (see Supplementary Table S1 and Figure 4). The hierarchical grouping of the DEGs illustrated in a heatmap represents the level of expression in each replicate sample when comparing the virulent strain vs. the attenuated strain, allowing for the detection of genes with a similar change in expression (Supplementary Figure S6). This type of map enables the visualization of each gene (rows) for each of the biological triplicates distinguishing between the virulent strain and the attenuated strain (columns). Higher expression levels are represented in yellow, while lower expression is depicted in blue, based on the Z score. By performing a filtering of the differentially expressed genes, the top 100 DE genes were selected, which can be viewed on the heatmap (Supplementary Figure S6). Out of these 100 genes, 41 correspond to *Babesia bigemina* hypothetical protein, partial mRNA, 29 to *B. bigemina* ribosomal proteins, 7 to *B. bigemina* histones, putative partial mRNA, 5 to *B. bigemina* translation initiation, putative partial mRNA, and the remaining 18 genes to other genes. 

### 3.5. In Silico Analysis of Genes with Importance in Apicomplexa Organisms

Of the 100 genes with the highest significance (Supplementary Figure S6), a manual in silico analysis using the NCBI database results showed that 38 up-regulated genes encoding proteins of unknown function were found in the virulent strain and 3 up-regulated genes in the attenuated strain. BlastP homology analysis demonstrated that of the 38 genes up-regulated in the virulent strain and the 3 up-regulated in the attenuated strain, 35 and 3 genes have significant sequence identity with *Babesia* spp. genes in the virulent and attenuated strains, respectively (Supplementary Table S2).

### 3.6. Gene Ontology Enrichment

Out of the total number of differentially expressed genes, it was possible to identify that 482 correspond to up-regulated genes, and 37 genes are down-regulated, when comparing the virulent strain to the attenuated strain. Regarding the categorization of terms for upregulated genes, 21 terms were associated with Biological Processes, 6 with Cellular Components, and 4 with Molecular Functions (Table 2). A directed acyclic graph (DAG) depicts the results of a GO enrichment analysis of DEGs and indicates the containment relationships between terms (Supplementary Figure S7). Regarding down-regulated genes, the categorization of the gene ontology enrichment classified 16 terms belonging to Biological Processes. The number of DEGs assigned to the Cellular Component category in a GO enrichment analysis was 9, whereas it was 10 in Molecular Functions (Table 3). The category associated with biological processes most frequently found was GO:0006412 translation; however, the genes possibly associated with virulence, following the DAG in the three categories, correspond to XM_012910993.1 Protein kinase domain-containing protein, putative; and XM_012913418.1 60S acidic ribosomal protein, which were down-regulated. On the other hand, genes XM_012913282.1 TBC1 domain family member GTPase-activating protein, putative; XM_012910606.1 hypothetical protein; XP_012770178 RuvB-like 2 DNA helicase, putative; and XM_012914699.1 MORN repeat domain-containing protein, putative, were the overexpressed genes.

### 3.7. Comparative Analysis in Differential Gene Expression of a Virulent Strain vs. an Attenuated Strain of B. bigemina

The results of the differential expression and GO analyses allowed us to identify some of the genes of interest showing gene down-regulation (−1.9 to −3 LogFC) and up-regulation (2.4 to 2.9 LogFC), which might be associated with virulent factors in *B. bigemina*. Worth noting for genes with up-regulation are the 12d3 antigen, putative XM_012910910.1; cAMP-dependent protein kinase regulatory subunit (XM_012910910.1); putative protein kinase domain-containing protein (XM_012910993.1 and XM_012913697.1); putative Ras family protein (XM_012913885.1); and acid phosphatase, putative (XM_012914848.1); which were particularly overexpressed in the virulent strain as opposed to the attenuated strain. By contrast, the down-regulated genes included a hypothetical protein (XM_012910606.1); GTPase-activating protein of the TBC1 domain family member (XM_012913282.1); and protein containing the MORN repeat domain (XM_012914699.1) in the virulent strain (Table 4).

## 4. Discussion

### 4.1. Obtaining Biological Material

To obtain the biological material of the *Babesia bigemina* virulent strain, a splenectomy was performed on a *Bos taurus* calf to reactivate the virulent parasite population, which was inoculated with a cryostabilate kept in liquid nitrogen. It is important to mention that this surgical technique is commonly used to recover *Babesia* parasites that have been maintained in liquid nitrogen for a prolonged period of time [8]. Previously, it has also been used as an attenuation procedure in the Babesia field of research. For example, successive passages of *B. bovis* have been reported to result in progressively less severe signs of disease and reduced virulence in splenectomized calves [35]. Likewise, this previously described method has been used to develop a live attenuated vaccine by attenuating *Babesia* strains in splenectomized calves [1,6,7,35]. The results obtained during clinical monitoring showed that the steers inoculated with the virulent strain of *Babesia bigemina* presented severe clinical disease, as evidenced by the high parasitemia, reaching a maximum PPE of ≥8, a reduced PCV of 15%, and a rectal temperature >41 °C, required specific treatment against bovine babesiosis, in addition to using supportive therapy to reestablish them clinically. On the other hand, cattle inoculated with the attenuated strain, derived from in vitro culture, and previously tested as a live attenuated vaccine, showed moderate clinical signs, with maximum parasitemia reaching a PPE <0.1%, maximum rectal temperature values of 39.5 °C, and a slight decrease in PCV. Previous studies have evaluated the effectiveness of an attenuated vaccine derived from in vitro culture of live *Babesia bigemina* and *Babesia bovis*, demonstrating that when the vaccine parasites were established in inoculated cattle and challenged with virulent isolates, they generated adequately protective immunity, either under controlled pen experimental trials or through natural exposure to *Babesia*-infected ticks in the field [8,12,22,36,37]. The aforementioned is essential for this study since it is well-known that the attenuation of *Babesia bigemina* can be successfully achieved under in vitro culture conditions, leading to its use as a live attenuated vaccine. However, at the level of gene expression, it was not known until now what type of molecular events could be occurring in the *B. bigemina* virulent parasites as compared to those attenuated after a prolonged time under in vitro culture conditions. Thus, this study presents the first comparison of gene expression, using RNA-seq technology, between a virulent strain and an attenuated strain of *Babesia bigemina* from Mexico.

### 4.2. Sequencing and Bioinformatic Analysis

RNA Next Generation Sequencing (RNA-seq) provides unprecedented, detailed information about the transcriptional landscape of an organism and enables precise measurement of the expression level of transcripts in a sample [38]. The reference genome used in this study to map the obtained reads was that of the *B. bigemina* BOND (RefSeq: GCF_000981445.1). This reference genome was sequenced to 8x coverage using capillary technology and assembled with Phrap. The genome was manually repaired in Gap4. Sequencing errors were corrected by polymerase chain reaction, giving a *B. bigemina* genome size of 13.8 Mb, a Contig N50 size of 2.5 Mb, and 5080 genes [39]. In our study, despite a theoretical 75X coverage, mapping of the generated reads against the *B. bigemina* reference genome resulted in a *B. bigemina* transcriptome containing only 3544 genes of the total *B. bigemina* gene content. Due to the variation between biological replicates, especially for an attenuated strain on a multidimensional scale, it has been suggested that specific transcriptional responses to parasite infection may be difficult to detect in natural populations with high individual genetic diversity. Thus, the importance of transcriptional variation in response to specific interactions between host and parasite genotypes [40]. Furthermore, host–parasite evolutionary dynamics in many systems have led to the emergence and maintenance of different parasite and host genotypes within the same population. Genotypes differ in fundamental characteristics: parasite genotypes differ in their infectivity, host genotypes differ in susceptibility, and the outcome of infection is often a consequence of the genotypic identity of both parties [41].

On the other hand, in a similar study, variability was identified in biological replicates by comparing blood stages of *B. bovis* in the host vs. kinete stages in the vector, examining their gene expression. Principal component analysis (PCA) showed that the pattern of gene expression in the *B. bovis* kinete samples was different from the blood phase samples. The first principal component explained 90% of the variation in the data. Furthermore, PCA shows that the transcriptomes of the *B. bovis* blood stages are similar to each other as compared to the transcripts from the *B. bovis* kinete samples, as shown by the stronger clustering of the blood stage samples in the second principal component [42]. In the bioinformatics analysis performed, at least 519 differentially expressed genes were detected when the transcriptome of the *B. bigemina* virulent strain was compared against the attenuated strain. *Babesia* parasites were obtained during the clinical phase from the virulent strain and in the establishment of the vaccine parasites of the attenuated strain (both parasite populations obtained from intact cattle with spleen, and without purposive immunosuppression). Out of the 519 DEGs, 482 corresponded to genes being up-regulated, whereas 37 genes were down-regulated. The variability generated in the reads obtained between the biological triplicates of the virulent strain obtained by RNA-seq analysis could be associated with the fact that some pathogens manage to adapt dynamically and continuously to a susceptible host. This can generate variability in virulence at the population level, which can also vary due to the genetic make-up of the host and the host’s immunological status, among other features. 

### 4.3. In Silico Homology Analysis of Genes with Other Apicomplexa Organisms

In this study, several up-regulated genes that encode hypothetical proteins were found in the virulent strain. With the in silico analysis performed, a high percentage of sequence identities were identified with annotated genes, especially those of *Babesia ovata*. It is a bovine *Babesia* species originally isolated from Japan, transmitted by the ixodid tick *Haemaphysalis longicornis*, and widely distributed in several East Asian countries [43,44]. Clinical signs in cattle infected with *B. ovata* are usually mild, including fever and anemia [45]. In addition to *B. ovata*, several species of *Babesia* can infect livestock. Phylogenetic and evolutionary analyses using 18S rRNA sequences show that *B. bigemina* is the closest species to *B. ovata*. However, clinical signs are more severe in cattle infected with *B. bigemina* than in those infected with *B. ovata* [1,43,46]. With regard to the second species, in which a high sequence identity of *B. bigemina* DEGs was identified, that species is *B. caballi*. Previous studies have shown that there are at least 263 orthologous genes that have been identified in ten different species of the Apicomplexa (i.e., *B. bovis*, *B. bigemina*, *B. ovata*, *B. microti*, *B. divergens*, *Babesia* sp. *Xinjiang*, *T. equi*, *P. falciparum*, *T. gondii* and *B. caballi*). Finally, it is important to note that *B. caballi* was phylogenetically closer to *B. bigemina* and *B. ovata* than to *B. bovis* [47].

### 4.4. Gene Ontology Enrichment

Bovine babesiosis is a disease that affects cattle in the tropical and subtropical regions of Mexico and around the world [1]. Currently, the only prototype of a live attenuated vaccine in Mexico has been developed at CENID-SAI, INIFAP [8]. Thus, it was of utmost importance and interest to identify the differentially expressed genes between a virulent parental *B. bigemina* strain and an attenuated in vitro culture-derived *B. bigemina* strain, which have been subject to study as a live attenuated vaccine during the last 30 years [8]. RNA-seq followed by a bioinformatic differential expression analysis could help identify differences in the total gene expression existent between a virulent parasite population and the attenuated in vitro culture-derived *B. bigemina* strain. In this way, and taking advantage of the GO classification, a gene enrichment bioinformatic procedure can be used to analyze the performance and categorization of the results obtained in the differential expression analysis. Thus, by performing the GO enrichment analysis, and based on the subgraph produced by the R program “TopGO” (v2.46.0), we selected some enrichment terms and their corresponding genes that could possibly be implicated in the virulent phenotype of a wild type *B. bigemina* strain, and/or in the attenuation features of the *B. bigemina* strain used as a live attenuated vaccine. Thus, from the group of up-regulated genes specifically identified in the virulent strain of *B. bigemina* and with a biological process classification, the term GO:0032147 activation of protein kinase, the putative protein kinase domain-containing protein genes (XM_012913697 and XM_012910993) are highlighted. In this context, a previous study on the genomic comparison of these same virulent and attenuated strains of *B. bigemina* resulted in the identification of 27 virulence-associated genes, out of which only 5 were definitely identified in the attenuated strain, including a calcium-dependent protein kinase 4 (XM_012911530.1), Calmodulin-domain protein kinase 2 (XM_012910710.1), cAMP-dependent protein kinase (XM_012914446.1), and Casein kinase I (XM_012913338.1), to name a few, enzymes that belong to the kinase family of proteins [21]. Biologically, proteins of the kinase family are fundamental in some signaling pathways of apicomplexans, including *Babesia* spp. Kinases are essential for the invasion of the parasite, in addition to the ligands of the parasite to the cell. Likewise, kinases comprise a class of Apicomplexa-specific serine/threonine enzymes known as calcium-dependent protein kinases (CDPKs), involved in cellular processes such as glidding, as well as invasion, mainly emphasizing the role of phosphorylation and calcium-based signaling. On the other hand, it is important to mention that, in *Babesia bovis*, 44 protein kinases have been reported [48]. This information is very important since, in this study, they are overexpressed in the virulent strain, unlike the attenuated strain. These molecules are important as possible promising pharmacological targets in the future for parasitic diseases caused by apicomplexans, despite the fact that these kinases have structural differences [49]. On the other hand, the kinase domain-containing protein has been studied in *Plasmodium*, where several functions for the PKA (dependent protein kinase) in the pathogenesis of malaria have been defined. The recently described *Plasmodium falciparum* phosphoproteomes introduced a large volume of phosphopeptide data for both basic research and the identification of new therapeutic targets against malaria. Phosphorylation at sites in the activation cycle could be mediating several processes, from regulating parasite kinase activity to mediating the coupling of other proteins. Important differences between *Plasmodium* and mammalian PKA isoforms indicate that the parasite kinase is a valid therapeutic target against malaria [50] and, perhaps, in *Babesia*.

Within the genes that were overexpressed in the category of biological processes, the term GO:0051250, defined as negative regulation of lymphocyte activation, was identified in the virulent strain vs. the attenuated strain. In a study where a comparison of a *Babesia bovis* virulent strain and an attenuated strain was performed by a microarray analysis with two biological replicas, a total of 78% and 89% detectable transcripts were found, identifying differentially regulated transcripts within each pair of strains [51]. These differentially regulated transcripts included *VESA1*, *SmORFs*, undefined membrane and hypothetical protein-coding genes. It was reported that the majority of strain-regulated individual specific gene transcripts were not shared between the two strains [51]. Other studies with *B. bovis* have also shown upregulation of calcium-dependent protein kinase 4 (*cdpk4*), tubulin-tyrosine ligase (*ttl*), and methyltransferase (*mt*) genes in sexually induced parasite stages in vitro and parasite development in its vector tick [52,53]. 

Another term classified within the biological processes possibly associated with virulence and up-regulated in the *B. bigemina* virulent strain is GO:0042742 defense response to the bacteria, which manages to find the *12d3* gene, a parasite antigen originally described in *B. bovis*. This gene has been previously identified as being expressed in *B. bigemina* parasites derived from in vitro culture [54] and it is a highly conserved *12d3* gene in 20 Mexican isolates of *Babesia bovis* [55]. Within the results of the up-regulated genes identified in this study and related to the defense response to bacterium biological process category, the Ras family protein putative (XP_012769339) is worth highlighting. Ras superfamily signaling depends on the binding of specific effectors. Therefore, small changes in the sequence, structure, and/or cellular regulation of superfamily members affect binding to regulators and, thus, cellular signaling. The Ras superfamily is divided into five main families: Ras, Rho, Arf/Sar, Ran, and Rab [56]. Members of the Ras family function as signaling hubs activated by various extracellular mechanisms that stimulate and regulate intracellular signaling. Rab genes code for small GTP-binding proteins of the Ras superfamily. These proteins contribute to the targeting and fusion of transport vesicles in the secretory and endocytic pathways [56,57]. A family of Rab GTPases has been identified in *Plasmodium*. Additionally, most Apicomplexa parasites share common signal peptides of the secretory pathway proteins. This signaling ultimately controls gene transcription, which in turn affects fundamental processes such as cell growth and differentiation [58]. There is information on a phylogenetic analysis that indicated a core set of essential Rabs in Apicomplexan parasites, with *Babesia bovis* encoding 9 Rabs, as well as *Theileria annulata* and *T. parva* [58]. 

The last gene selected from the biological processes category term is an acid phosphatase putative (XP_012770302). The site of acid phosphatase activity is known to occur in the erythrocytic phase of *Plasmodium gallinaceum* and *P. berghei*. Acid phosphatase activity has been demonstrated in the food tubes and endoplasmic reticulum of both parasites [59]. This study identified the food vacuole as the site of digestion in the host cell cytoplasm. In addition, enzyme activity has been observed in compartments or structures that are located in infected host cells. Apparently, this mechanism does not exist in *B. bigemina*, as the parasite does not generate a vacuole similar to that found in *Plasmodium*. However, in *B. microti*, while it lacks a parasitophorous vacuole, there is evidence that after the invasion of the host erythrocyte, it undergoes an important morphogenetic change during which it produces an intertwining of vesicles, representing a new mechanism for the delivery of parasitic factors to the host in the *phylum Apicomplexa* [60]. 

Within the classification of cellular components, the term GO:0005952 includes genes that encode for proteins such as the cAMP-dependent protein kinase complex; cAMP-dependent protein kinase regulatory subunit, putative (XP_012766364), and protein kinase domain-containing protein, putative (XP_012766447), and those characterized by the AGC family of serine–threonine protein kinases as the effector kinase of cAMP signaling. In this manner, protein kinase A (PKA) is involved in the regulation of a variety of cellular processes, including metabolism, cell development, gene expression, and apoptosis. The cAMP-dependent PKA signaling pathway plays a vital part amid the pathogenesis and virulence of various pathogens. Since cAMP flux is involved in numerous intracellular processes, the PKA signaling pathway may affect various pathological inflammatory processes. In addition, cAMP–PKA signaling pathways have been identified that are relevant to *Plasmodium falciparum* infection of erythrocytes, as well as a kinase anchor protein (AKAP) targeting PKA in PGE2 signaling through the prostaglandin receptor for PTGER4 (EP4)-PKA prostaglandin signaling in *Toxoplasma* [61].

With regard to the gene TBC1 domain family member GTPase-activating protein putative (XM_012913282), data on GTPases from apicomplexans are available, indicating that they are involved in the infection process of intracellular parasites. Three GTPases, Rac1, Cdc42 and Arf6, have been found to be involved in the invasion of host cells by *Trypanosoma cruzi*. During invasion, these GTPases promote parasite penetration by regulating the actin cytoskeleton at the site of parasite invasion. A similar mechanism has been observed in the invasion of the intracellular parasite *Leishmania donovani*, where the Rac1 and Arf6 genes are activated when the parasite enters macrophages and mediates phagocytosis. After invasion, Rac1 localizes to the phagosome where *L. donovani* resides, where it interacts with Cdc42 to form an F-actin sheath around the phagosome, thereby inhibiting phagosome maturation [62].

Recently, in studies on the avian influenza virus (IAV), where a multi-omic analysis was performed, a subset of IFN-dependent and independent cellular defense mechanisms that inhibit IAV replication have been identified. Among them, the autophagy regulator TBC1 domain family member 5 (TBC1D5) is highlighted. TBC1D5 binds to Rab7 to allow autophagosome–lysosome fusion, regulating IAV replication in vitro and in vivo and promoting IAV lysosomal replication by targeting the M2 protein of the influenza virus [63]. Likewise, the eukaryotic protein kinase G PknG promotes the induction of macroautophagy/autophagy but inhibits the maturation of the autophagosome, as reported in *Mycobacterium tuberculosis*, generating the effect of blocking the flow of autophagy and increasing intracellular survival of the pathogen [64]. PknG inhibits the activation of AKT (AKT serine/threonine kinase) by competitively binding to its pleckstrin (PH) homology domain, causing the induction of autophagy. PknG can also inhibit autophagosome maturation to inhibit autophagic flux by targeting RAB14, a host small GTPase. Importantly, PknG directly interacts with RAB14 and inhibits RAB14–GTP hydrolysis. In addition, PknG inhibits GTPases by phosphorylating TBC1D4/AS160 (TBC1 domain family member 4). Taken together, these results reveal a dual-function bacterial effector that tightly regulates host autophagy flux to benefit intracellular pathogen survival [65]. Whether this or a similar mechanism might be occurring in *Babesia bigemina* remains to be tested. 

Finally, the term GO:0016308, 1-phosphatidylinositol-4-phosphate 5-kinase activity, with a classification based on molecular function, selected the MORN repeat domain-containing protein, putative gene (XP_012770153). Currently, not much is known about this protein in apicomplexan organisms; studies performed with *Toxoplasma gondii* identified proteins with multiple membrane localization and assembly (MORN) motifs. MORN1 appears to be conserved among apicomplexans. Interestingly, MORN1 is specific to centrosomes, specialized nuclear structures that form the spindle and ring structures at the apical and posterior ends of the endomembrane complex [66]. BEWA_033160 was annotated as a MORN repeat domain-containing protein in the genome of *Theileria equi* and was identified as a member of a cluster of genes orthologous to *Plasmodium*, *Theileria*, *B. bovis*, *Toxoplasma gondii*, and *Cryptosporidium parvum* proteins [67].

No doubt further experimental work needs to be done in *B. bigemina* to demonstrate that the regulated gene transcripts identified in this study are involved in some sort of mechanism participating in the virulence/attenuation expression phenotype of the *B. bigemina* parasite populations analyzed. In addition, steers were used in this particular study due to their availability and cost. One-year-old steers are as susceptible as older animals. Previous studies, where the attenuated strain (live attenuated vaccine) was used, have included steers, bulls, cows and pregnant cows [8]. In all cases, 80 to 100% efficacy has been obtained when the attenuated *Babesia* parasites are used as a vaccine. Further studies are required to determine if the DEGs identified in this study would also be found differentially expressed in heifers, cows and/or older bulls infected with virulent *B. bigemina* parasites. 

## 5. Conclusions

The findings presented in this study offer a pioneering comparison of the transcriptomes between a virulent and an attenuated *Babesia bigemina* strain of Mexican origin using RNA-seq through Illumina sequencing. The data generated provides essential information to better understand the biological processes that exist for each of the *Babesia bigemina* strains used in this study. This will eventually allow for the identification and targeting of specific genes to design precise intervention strategies, such as the development of new diagnostic tools, vaccines, and innovative pharmaceutical drugs for the control of bovine babesiosis caused by *B. bigemina*. The potential impact of these findings holds promise for advancing both scientific knowledge and practical solutions in the field.

## Figures and Tables

**Table 1:** Summary of the sequencing results for three biological replicates of the Mexican *Babesia bigemina* virulent and attenuated strains, with paired-end readings.

	Replicate ID.	Total Number of Reads	Length of Read (Bases)	% GC
Virulent strain	15026_S1_R1_001	15,725,738	76	53
	15026_S1_R2_001	15,725,738	76	53
	15027_S2_R1_001	13,426,598	76	53
	15027_S2_R2_001	13,426,598	76	53
	15028_S3_R1_001	13,756,826	76	53
	15028_S3_R2_001	13,756,826	76	53
Attenuated strain	15029_S4_R1_001	9,512,321	76	53
	15029_S4_R2_001	9,512,321	76	53
	15030_S5_R2_001	9,504,042	76	53
	15030_S5_R2_001	9,504,042	76	53
	15031_S6_R2_001	9,656,042	76	53
	15031_S6_R2_001	9,656,042	76	53

**Table 2:** Significantly enriched GO terms in the up-regulated transcripts for the attenuated strain ranked according to the *p*-value < 0.05.

Term		Annotated	Significant	Expected	*p*-Value
Biological Process Terms					
GO:0032502	Developmental process	229	5	2.07	0.016
GO:0019751	Polyol metabolic process	8	2	0.07	0.026
GO:0019919	Peptidyl-arginine methylation, to asymmetrical-dimethyl arginine	3	1	0.03	0.027
GO:0001819	Positive regulation of cytokine production	3	1	0.03	0.027
GO:0051353	Positive regulation of oxidoreductase activity	3	1	0.03	0.027
GO:0055129	L-proline biosynthetic process	3	1	0.03	0.027
GO:0034969	Histone arginine methylation	3	1	0.03	0.027
GO:0043583	Ear development	3	1	0.03	0.027
GO:0051250	Negative regulation of lymphocyte activation	3	1	0.03	0.027
GO:0090407	Organophosphate biosynthetic process	120	4	1.09	0.032
GO:0090596	Sensory organ morphogenesis	4	1	0.04	0.036
GO:0009846	Pollen germination	4	1	0.04	0
GO:0032103	Positive regulation of response to external stimulus	4	1	0.04	0.036
GO:0055123	Digestive system development	4	1	0.04	0.036
GO:0042364	Water-soluble vitamin biosynthetic process	4	1	0.04	0.036
GO:0051607	Defense response to virus	4	1	0.04	0.036
GO:0048568	Embryonic organ development	5	1	0.05	0.044
GO:0010565	Regulation of cellular ketone metabolic process	5	1	0.05	0.044
GO:0006071	Glycerol metabolic process	5	1	0.05	0.044
GO:0009860	Pollen tube growth	5	1	0.05	0.044
GO:0006310	DNA recombination	40	2	0.36	0.049
Cellular Components terms					
GO:0031011	Ino80 complex	3	1	0.02	0.023
GO:0031253	Cell projection membrane	3	1	0.02	0.023
GO:0000118	Histone deacetylase complex	3	1	0.02	0.023
GO:0030175	Filopodium	3	1	0.02	0.023
GO:0000794	Condensed nuclear chromosome	5	1	0.04	0.039
GO:0071339	MLL1 complex	5	1	0.04	0.039
Molecular Function terms					
GO:0008171	O-methyltransferase activity	3	1	0.03	0.026
GO:0008469	Histone-arginine N-methyltransferase activity	3	1	0.026	0.03
GO:0050661	NADP binding	4	1	0.04	0.035
GO:0016308	1-phosphatidylinositol-4-phosphate 5-kinase activity	5	1	0.04	0.043

“**Annotated**” stands for “the total number of times the GO is found in the reference”; “**Significant”** stands for “the total number of times the GO is found in the list of differentially expressed genes”; “**Expected**” stands for “the expected value by chance that the GO should be found in the list”.

**Table 3:** Significantly enriched GO terms in the down-regulated genes for the attenuated strain; transcripts ranked according to the *p*-value < 0.05.

GO Term		Annotated	Significant	Expressed	*p*-Value
Biological Process Terms					
GO:0006412	Translation	323	81	34.28	8.60 × 10^−17^
GO:0002181	Cytoplasmic translation	36	16	3.82	9.60 × 10^−8^
GO:0000028	Ribosomal small subunit assembly	9	6	0.96	8.60 × 10^−5^
GO:0006414	Translational elongation	36	11	3.82	0.0022
GO:0022900	Electron transport chain	20	6	2.12	0
GO:0042254	Ribosome biogenesis	208	31	22.08	0.0197
GO:0042742	Defense response to bacterium	7	3	0.74	0.0299
GO:0046034	ATP metabolic process	35	9	3.71	0.0303
GO:0030433	Ubiquitin-dependent ERAD pathway	12	4	1.27	0.0308
GO:0006913	Nucleocytoplasmic transport	37	6	3.93	0.0312
GO:1902236	Negative regulation of endoplasmic reticulum stress-Induced intrinsic apoptotic…	3	2	0.32	0.0313
GO:0008589	Regulation of smoothened signaling pathway	3	2	0.32	0.0313
GO:0034629	Cellular protein-containing complex localization	3	2	0.32	0.0313
GO:0032147	Activation of protein kinase activity	3	2	0.32	0.0313
GO:0045047	Protein targeting to ER	14	3	1.49	0.0313
GO:0000027	Ribosomal large subunit assembly	18	5	1.91	0.0348
Cellular Components terms					
GO:0022625	Cytosolic large ribosomal subunit	35	20	3.61	6.30 × 10^−12^
GO:0005840	Ribosome	171	66	17.63	5.30 × 10^−10^
GO:0000786	Nucleosome	9	8	0.93	9.30 × 10^−8^
GO:0022627	Cytosolic small ribosomal subunit	21	11	2.16	1.50 × 10^−6^
GO:0070469	Respirasome	12	5	1.24	0.0054
GO:0015935	Small ribosomal subunit	31	15	3.2	0.012
GO:0033180	Proton-transporting V-type ATPase, V1 domain	6	3	0.62	0.0294
GO:0005952	cAMP-dependent protein kinase complex	3	2	0.31	0.0296
GO:0005618	Cell wall…	3	2	0.31	0.0296
Molecular Function terms					
GO:0003735	Structural constituent of ribosome	132	58	13.9	3.10 × 10^−25^
GO:0046982	Protein heterodimerization activity	29	11	3.05	8.60 × 10^−5^
GO:0020037	Heme binding	6	4	0.63	0.0015
GO:0046961	Proton-transporting ATPase activity, rotational Mechanism	14	5	1.47	0.0112
GO:0019843	rRNA binding	61	15	6.42	0.0113
GO:0003746	Translation elongation factor activity	15	5	1.58	0.0153
GO:0045296	Cadherin binding	11	4	1.16	0.0218
GO:0008097	5S rRNA binding	3	2	0.32	0.0308
GO:0015450	Protein-transporting ATPase activity	3	2	0.32	0.0308
GO:0009055	Electron transfer activity	18	5	1.9	0.0337

“**Annotated**” stands for “the total number of times the GO is found in the reference”; “**Significant”** stands for “the total number of times the GO is found in the list of differentially expressed genes”; “**Expected**” stands for “the expected value by chance that the GO should be found in the list”.

**Table 4:** Up-regulated and down-regulated genes of attenuated versus virulent strains of *B. bigemina*, with possible association with one or more virulent factors. Cut-off is log 2-fold change in expression, *p*-value ≤ 0.01, (n = 3).

Gene ID (NCBI)	Gene	logFC	*p*-Value	Regulation
XM_012914699	MORN repeat domain containing protein, putative	2.476133206	0.000475261	DOWN_virulent_UP_attenuated
XM_012910606	Hypothetical protein	2.56427365	0.007392035	DOWN_virulent_UP_attenuated
XM_012913282	TBC1 DOMAIN FAMILY MEMBER GTPASE-ACTIVATING PROTEIN, putative	2.913638058	0.002352895	DOWN_virulent_UP_attenuated
XM_012913697	Protein kinase domain containing protein, putative	−1.98020918	0.00151951	UP_virulent_DOWN_attenuated
XM_012913885	Ras family protein, putative	−2.2238797	0.000341439	UP_virulent_DOWN_attenuated
XM_012914848	Acid phosphatase, putative	−2.52697756	0.001797584	UP_virulent_DOWN_attenuated
XM_012910976	12D3 antigen, putative	−2.99076308	0.000124446	UP_virulent_DOWN_attenuated
XM_012910910	cAMP-dependent protein kinase regulatory subunit, putative	−3.1032439	0.000142516	UP_virulent_DOWN_attenuated
XM_012910993	Protein kinase domain containing protein, putative	−3.37794128	0.000194011	UP_virulent_DOWN_attenuated

## Data Availability

Data are contained within the article and Supplementary Material. Nucleotide sequence data reported in this paper are available in the NCBI (https://www.ncbi.nlm.nih.gov/bioproject/?term=PRJNA1038707) (accessed on 10 November 2023) database under the accession number BioProject ID: PRJNA1038707.

## Supplementary Materials

The following supporting information can be downloaded at: https://www.mdpi.com/article/10.3390/vaccines12030309/s1. Figure S1: Rectal temperature (mean ± SD) in cattle inoculated with a virulent and attenuated strain of B. bigemina. GI: Virulent strain (Blue line) and GII: Attenuated strain (Orange line); Figure S2: Monitoring of Packed Cell Volume (PCV) using the microhematocrit technique (mean ± SD) in cattle inoculated with a virulent or an attenuated strain of B. bigemina. GI: Virulent strain (Blue line) and GII: Attenuated strain (Orange line); Figure S3: Percoll density gradient concentration at 68.46%. (A) Intermediate phase, erythrocytes parasitized with trophozoites and merozoites of B. bigemina are observed. (B) Lower phase, mononuclear cells (MC) and free trophozoites/merozoites intermingled with the MC. Giemsa-stained smear, 100×; Figure S4: Multidimensional scale (MDS) and Normalized TMM counts graphs where the distance between samples and conditions reflects their similarity, for each biological sample of the attenuated and virulent strain of B. bigemina; Figure S5: CPM Plot. A bar plot for each sample is generated where Counts Per Million for each gene in each biological sample are represented in the attenuated and virulent strains of B. bigemina; Figure S6: Heatmap of the differentially expressed genes in the virulent strain vs. the attenuated strain of Babesia bigemina. A higher level of expression can be observed in yellow and a lower expression in blue, according to a Z score (right legend of the map); Figure S7: Directed acyclic graph (DAG) depicting the results of a GO enrichment analysis of DEGs; Table S1: Differentially expressed genes identified between the virulent strain and the attenuated strain of Babesia bigemina in Mexico; Table S2: B. bigemina virulent strain Up-regulated genes.

## References

[1] Bock R.E., Jackson L., De Vos A.J., Jorgensen W. (2004). Babesiosis of cattle. Parasitology.

[2] Bautista G.C., Martínez I.F., Woldemeskel M. (2012). Experiences on the control of cattle tick *Rhipicephalus (Boophilus) microplus* in Mexico. Ticks: Disease, Management and Control.

[3] Rodríguez V.R.I., Grisi L., Pérez L.A.A., Silva V.H., Torres A.J.F., Fragoso S.H., Romero S.D., Rosario C.R., Saldiernah F., García C.D. (2017). Potential economic impact assessment for cattle parasites in Mexico. Review. Rev. Mex. Cienc. Pecu..

[4] OIE World Organization for Animal Health (WOAH) (2021). Bovine Babesiosis.

[5] Alzan H.F., Knowles D.P., Suarez C.E. (2016). Comparative bioinformatics analysis of transcription factor genes indicates conservation of key regulatory domains among *Babesia bovis*, *Babesia microti*, and *Theileria equi*. PLoS Negl. Trop. Dis..

[6] de Vos A.J., Jorgensen W.K. (1992). Protection of cattle against babesiosis in tropical and subtropical countries with a live, frozen vaccine. Tick Vector Biology.

[7] Florin-Christensen M., Suarez C., Rodríguez A., Flores D., Schnittger L. (2014). Vaccines against bovine babesiosis: Where we are now and possible roads ahead. Parasitology.

[8] Alvarez J.A., Rojas C., Figueroa J.V. (2020). An overview of current knowledge on in vitro *Babesia* cultivation for production of live attenuated vaccines for bovine babesiosis in Mexico. Front. Vet. Sci..

[9] McElwain T.F., Palmer G.H., Goff W.L., McGuire T.C. (1988). Identification of *Babesia bigemina* and *Babesia bovis* merozoite proteins with isolate- and species-common epitopes recognized by antibodies in bovine immune sera. Infect. Immun..

[10] Figueroa J.V., Precigout E., Carcy B., Gorenflot A. (2006). Identification of common antigens in *Babesia bovis*, *B. bigemina*, and *B. divergens*. Ann. N. Y. Acad. Sci..

[11] Wright I.G., Goodger B.V., Leatch G., Aylward J.H., Rode-Bramanis K., Waltisbuhl D.J. (1987). Protection of *Babesia bigemina*-immune animals against subsequent challenge with virulent *Babesia bovis*. Infect. Immun..

[12] Vega C.A., Figueroa M.J.V., Rojas R.E., Ramos A.J.A., Cantó A.G. (1999). Insuficiente inmunidad cruzada en bovinos por *Babesia bigemina* y *Babesia bovis* derivadas de cultivo in vitro. Tec. Pecu. Méx..

[13] Figueroa J.V., Cantó G.J., Álvarez J.A., Loza G.R., Ramos J.A., Vega C.A. (1998). Capacidad protectora en bovinos de una cepa de *Babesia bigemina* derivada del cultivo in vitro. Tec. Pecu. Méx..

[14] Schetters T.P., Kleuskens J.A., Scholtes N.C., Gorenflot A., Moubri K., Vermeulen A.N. (2021). Vaccination of dogs against heterologous *Babesia canis* infection using antigens from culture supernatants. Vet. Parasitol..

[15] Montenegro S.J., Toro M., Leon E., Lopez R., Ristic M. (1987). Bovine babesiosis: Induction of protective immunity with culture-derived *Babesia bovis* and *Babesia bigemina* immunogens. Parasitol. Res..

[16] Rathinasamy V., Poole W.A., Bastos R.G., Suarez C.E., Cooke B.M. (2019). Babesiosis Vaccines: Lessons Learned, Challenges Ahead, and Future Glimpses. Trends Parasitol..

[17] Jerzak M., Gandurski A., Tokaj M., Stachera W., Szuba M., Dybicz M. (2023). Advances in Babesia Vaccine Development: An Overview. Pathogens.

[18] Rojas R.E.E., Juan Joel Mosqueda G.J.J., Álvarez M.J.A., Hernández O.R., Ramos A.J.A., Cantó A.G.J., Vega M.C.A., Figueroa M.J.V. (2011). Transmissibility of *Babesia bigemina* and *Babesia bovis* attenuated strains by *Rhipicephalus (Boophilus) microplus* ticks. Rev. Mex. Cienc. Pecu..

[19] Vega C.A., Buening G.M., Green T.J., Carson C.A. (1985). In vitro cultivation of *Babesia bigemina*. Am. J. Vet. Res..

[20] Vega C.A., Buening G.M., Rodriguez S.D., Carson C.A., McLaughlin K. (1985). Cryopreservation of *Babesia bigemina* for in vitro cultivation. Am. J. Vet. Res..

[21] Sachman R.B., Lozano L., Lira J.J., Martínez G., Rojas C., Álvarez A., Figueroa J.V. (2021). Comparative genomic study of attenuated and virulent strains of *Babesia bigemina*. Pathogens.

[22] Rojas-Martinez C., Rodriguez-Vivas R.I., Figueroa-Millan J.V., Acosta-Viana K.Y., Gutierrez-Ruiz E.J., Bautista-Garfias C.R., Lira-Amaya J.J., Polanco-Martinez D.J., Alvarez-Martinez J.A. (2018). *Babesia bigemina*: Advances in continuous in vitro culture using serum-free medium supplemented with insulin, transferrin, selenite, and putrescine. Parasitol. Int..

[23] Rojas-Martínez C., Rodríguez-Vivas R.I., Figueroa-Millán J.V., Bautista-Garfias C.R., Castañeda-Arriola R.O., Lira-Amaya J.J., Urióstegui-Vargas P., Carrasco-Ojeda J.J., Alvarez-Martínez J.A. (2018). Bovine babesiosis: Cattle protected in the field with a frozen vaccine containing *Babesia bovis* and *Babesia bigemina* cultured in vitro with a serum-free medium. Parasitol. Int..

[24] Vega C.A., Buening M.G., Rodriguez S.D., Carson C.A. (1986). Concentration and enzyme content of in vitro-cultured *Babesia bigemina*-infected erythrocyte. J. Protozool..

[25] Ausubel F.M., Brent R., Kingston R.E., Moore D.D., Seidman J.G., Smith J.A., Struhl K. (2000). Current Protocols in Molecular Biology.

[26] Schroederk A., Mueller O., Stocker S., Salowsky R., Leiber M., Gassmann M., Lightfoot S., Menzel W., Granzow M., Ragg T. (2006). The RIN: An RNA integrity number for assigning integrity values to RNA measurements. BMC Mol. Biol..

[27] Mueller O., Lightfoot S., Schroeder A. (2016). RNA Integrity Number (RIN)—Standardization of RNA Quality Control.

[28] Andrews S. (2010). FastQC: A Quality Control Tool for High Throughput Sequence Data. http://www.bioinformatics.babraham.ac.uk/projects/fastqc.

[29] Jimenez J.V., Sanchez F.A., Vega A.L. (2019). Integrative Differential Expression Analysis for Multiple EXperiments (IDEAMEX): A Web Server Tool for Integrated RNA-Seq Data Analysis. Front. Genet..

[30] Robinson M.D., McCarthy D.J., Smyth G.K. (2010). edgeR: A Bioconductor package for differential expression analysis of digital gene expression data. Bioinformatics.

[31] Anders S., Huber W. (2010). Differential expression analysis for sequence count data. Genome Biol..

[32] Tarazona S., García A.F., Dopazo J., Ferrer A., Conesa A. (2011). Differential expression in RNA-seq: A matter of depth. Genome Res..

[33] Altschul S.F., Gish W., Miller W., Myers E.W., Lipman D.J. (1990). Basic local alignment search tool. J. Mol. Biol..

[34] Alexa J.A., Rahnenfuhrer T.L. (2006). Improved scoring of functional groups from gene expression data by decorrelating GO graph structure. Bioinformatics.

[35] Callow L.L., Mellors L.T., McGregor W. (1979). Reduction in virulence of *Babesia bovis* due to rapid passage in splenectomized cattle. Int. J. Parasitol..

[36] Alvarez J.A., Ramos A.J., Rojas E., Mosqueda J.J., Vega M.C., Olvera A.M., Figueroa M.J., Canto A.G. (2004). Field challenge of cattle vaccinated with a combined *Babesia bovis* and *Babesia bigemina* frozen immunogen. Ann. N. Y. Acad. Sci..

[37] Bautista G.C.R., Lozano A.R., Rojas M.C., Álvarez M.J.A., Figueroa M.J.V., Reyes G.G., Castañeda A.R., Aguilar F.B.R. (2015). Co-immunization of cattle with a vaccine against babesiosis and Lactobacillus casei increases specific IgG1 levels to *Babesia bovis* and *B. bigemina*. Parasitology.

[38] Young M.D., Wakefield M.J., Smyth G.K., Oshlack A. (2010). Gene ontology analysis for RNA-seq: Accounting for selection bias. Genome Biol..

[39] Jackson A.P., Otto T.D., Darby A., Ramaprasad A., Xia D., Echaide I.E., Farber M., Gahlot S., Gamble J., Gupta D. (2014). The evolutionary dynamics of variant antigen genes in *Babesia* reveal a history of genomic innovation underlying host–parasite interaction. Nucleic Acids Res..

[40] Pawluk R.J., Uren W.T.M., Cable J., Garcia de Leaniz C., Consuegra S. (2018). Immune-Related Transcriptional Responses to Parasitic Infection in a Naturally Inbred Fish: Roles of Genotype and Individual Variation. Genome Biol. Evol..

[41] Barribeau S.M., Sadd B.M., Du Plessis L., Schmid H.P. (2014). Gene expression differences underlying genotype-by-genotype specificity in a host-parasite system. Proc. Natl. Acad. Sci. USA.

[42] Ueti M.W., Johnson W.C., Kappmeyer L.S., Herndon D.R., Mousel M.R., Reif K.E., Taus N.S., Ifeonu O.O., Silva J.C., Suarez C.E. (2021). Comparative analysis of gene expression between Babesia bovis blood stages and kinetes allowed by improved genome annotation. Int. J. Parasitol..

[43] Sivakumar T., Igarashi I., Yokoyama N. (2016). Babesia ovata: Taxonomy, phylogeny and epidemiology. Vet. Parasitol..

[44] Yoshinari T., Sivakumar T., Asada M., Battsetseg B., Huang X., Lan D.T.B., Inpankaew T., Ybañez A.P., Alhassan A., Thekisoe O.M. (2013). A PCR based survey of Babesia ovata in cattle from various. J. Vet. Med. Sci..

[45] Fujinaga T. (1981). Bovine babesiosis in Japan: Clinical and clinico-pathological studies on cattle experimentally infected with *Babesia ovata*. Nihon Juigaku Zasshi Jpn..

[46] Lack J.B., Reichard M.V., Van Den Bussche R.A. (2012). Phylogeny and evolution of the Piroplasmida as inferred from 18S rRNA sequences. Int. J. Parasitol..

[47] Ochi A., Kidaka T., Hakimi H., Asada M., Yamagishi J. (2023). Chromosome-level genome assembly of *Babesia caballi* reveals diversity of multigene families among *Babesia* species. BMC Genom..

[48] Elsworth B., Duraisingh M.T. (2021). A framework for signaling throughout the life cycle of *Babesia* species. Mol. Microbiol..

[49] Talevich E., Mirza A., Kannan N. (2011). Structural and evolutionary divergence of eukaryotic protein kinases in Apicomplexa. BMC Evol. Biol..

[50] Haste N.M., Talabani A., Doo A., Merckx A., Langsley G., Taylor S.S. (2012). Exploring the *Plasmodium falciparum* cyclic-adenosine monophosphate (cAMP)-dependent protein kinase (PfPKA) as a therapeutic target. Microbes Infect..

[51] Pedroni M.J., Sondgeroth K.S., Gallego L.G.M., Echaide I.E. (2013). Comparative transcriptome analysis of geographically distinct virulent and attenuated *Babesia bovis* strains reveals similar gene expression changes through attenuation. BMC Genm..

[52] Hussein H.E., Johnson W.C., Taus N.S., Capelli-Peixoto J., Suarez C.E., Mousel M.R., Ueti M.W. (2021). Differential expression of calcium-dependent protein kinase 4, tubulin tyrosine ligase, and methyltransferase by xanthurenic acid-induced *Babesia bovis* sexual stages. Parasit. Vectors.

[53] Harper G.S., Hibbs A.R., East I.J., Waltisbuhl D.J., Jorgensen W.K., Riddles P.W. (1996). *Babesia bovis*: Biosynthesis and localisation of 12D3 antigen in bovine erythrocytes. Int. J. Parasitol..

[54] de la Perez R.J.J., Vargas U.P., Álvarez M.J.A., Rojas M.C., González Z.V.M., Figueroa M.J.V. (2010). *Babesia bigemina*: Initial gene identification by Expressed Sequence Tags (EST) analysis of the intraerythrocytic stage. Rev. Mex. Cienc. Pecu..

[55] Perez J., Perez J.J., Vargas P., Alvarez J.A., Rojas C., Figueroa J.V. (2010). Sequence conservation of the 12D3 gene in Mexican isolates of *Babesia bovis*. Transbound. Emerg. Dis..

[56] Rojas M.A.M., Fuentes G., Valencia A. (2012). Evolution: The Ras protein superfamily: Evolutionary tree and role of conserved amino acids. J. Cell Biol..

[57] Karnoub A.E., Weinberg R.A. (2008). Ras oncogenes: Split personalities. Nat. Rev. Mol. Cell Biol..

[58] Langsley G., Van Noort V., Carret C., Meissner M., Villiers E.P., Bishop R., Pain A. (2008). Comparative genomics of the Rab protein family in Apicomplexan parasites. Microbes Infect..

[59] Sherman I.W. (1979). Biochemistry of *Plasmodium* (malarial parasites). Microbiol. Rev..

[60] Ben M.C., Thekkiniath J., Kilian N., Lawres L., Gewirtz M., Abraham A., Graham M., Liu X., Ledizet M. (2019). Parasite-derived vesicular-mediated protein export by the human pathogen *Babesia microti*. FASEB J..

[61] Haidar M., Ramdani G., Kennedy E.J., Langsley G. (2017). PKA and apicomplexan parasite diseases. Horm. Metab. Res..

[62] Paone S., Olivieri A. (2022). Role of host small GTPases in Apicomplexan parasite infection. Microorganisms.

[63] Sancho M., Tripathi L., Rodriguez F.S., Pache L., Sanchez A.M., McGregor M.J., Haas M.K., Swaney D.L., Nguyen T.T., João I. (2021). Restriction factor compendium for influenza A virus reveals a mechanism for evasion of autophagy. Nat. Microbiol..

[64] Van der Woude A.D., Stoop E.J., Stiess M., Wang S., Ummels R.M., Van Stempvoort G., Piersma S.R., Cascioferro A., Jiménez C.R., Houben E.N. (2014). Analysis of SecA2-dependent substrates in *Mycobacterium marinum* identifies protein kinase G (PknG) as a virulence effector. Cell Microbiol..

[65] Ge P., Lei Z., Yu Y., Lu Z., Qiang L., Chai Q., Zhang Y., Zhao D., Li B., Pang Y. (2022). Tuberculosis PknG manipulates host autophagy flux to promote pathogen intracellular survival. Autophagy.

[66] Gubbels M.J., Vaishnava S., Boot N., Dubremetz J.F., Striepen B. (2006). A MORN-repeat protein is a dynamic component of the *Toxoplasma gondii* cell division apparatus. J. Cell Sci..

[67] Kappmeyer L.S., Thiagarajan M., Herndon D.R., Ramsay J.D., Caler E., Djikeng A., Gillespie J.J., Lau A.O., Roalson E.H., Silva J.C. (2012). Comparative genomic analysis and phylogenetic position of *Theileria equi*. BMC Genom..

