# Supplementary material for: Comparative Transcriptome Analysis of Babesia bigemina Attenuated Vaccine and Virulent Strains of Mexican Origin
Source: Vaccines (Basel). 2024 Mar 15;12(3):309. doi: 10.3390/vaccines12030309 (PMC10975891; doi:10.3390/vaccines12030309)
Supplement: Supplementary file 1 [file vaccines-12-00309-s001.zip › Supplementary1_5RMSE.pdf]

Supplementary Figures

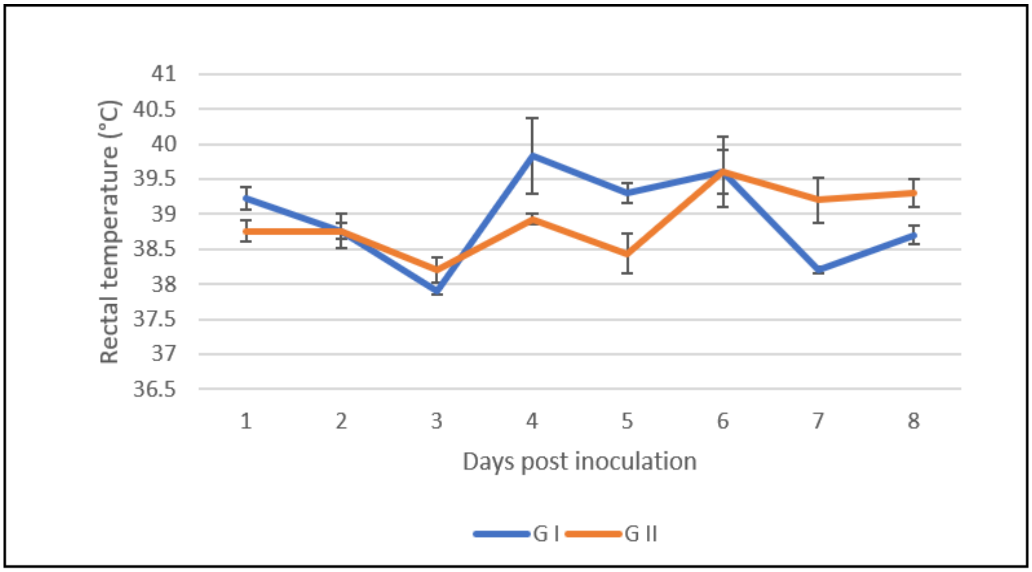

Supplementary Figure S1. Rectal temperature (mean ± SD) in cattle inoculated with a virulent and attenuated strain of *B. bigemina*. GI: Virulent strain (Blue line) and GII: Attenuated strain (Orange line).

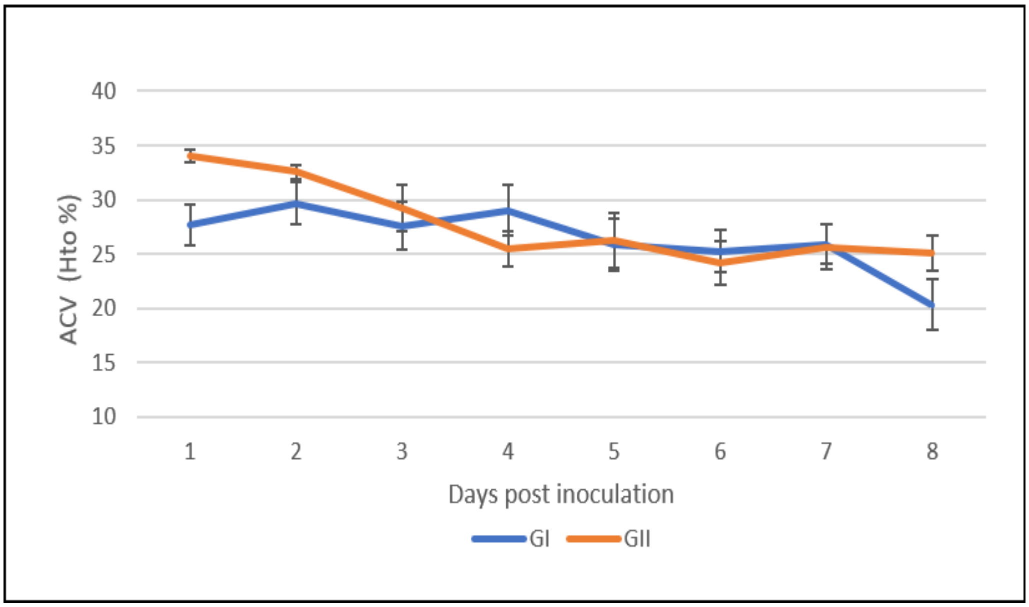

Supplementary Figure S2. Monitoring of Packed Cell Volume (PCV) using the microhematocrit technique (mean ± SD) in cattle inoculated with a virulent or an attenuated strain of *B. bigemina*. GI: Virulent strain (Blue line) and GII: Attenuated strain (Orange line).

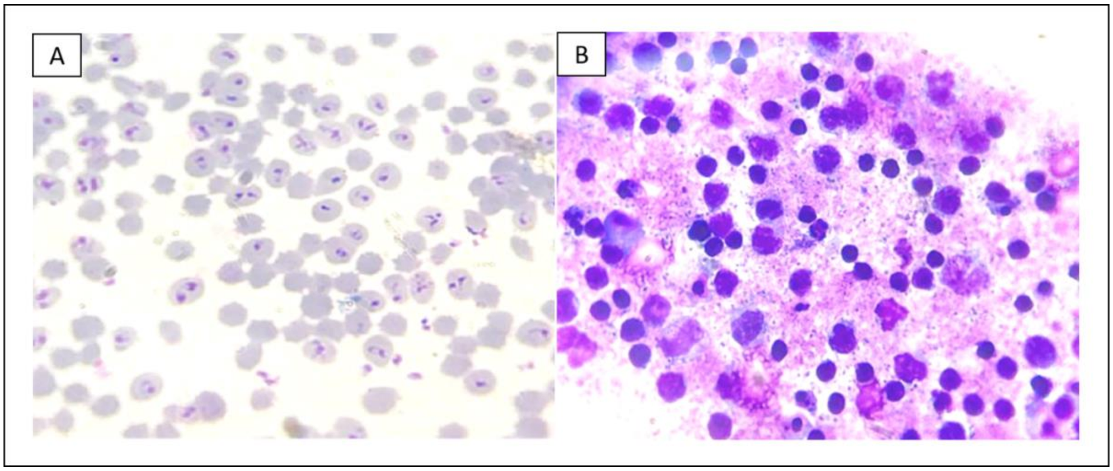

Supplementary Figure S3. Percoll density gradient concentration at 68.46%. A) Intermediate phase, erythrocytes parasitized with trophozoites and merozoites of *B. bigemina* are observed. B) Lower phase, mononuclear cells (MC) and free trophozoites/merozoites intermingled with the MC. Giemsa-stained smear, 100X.

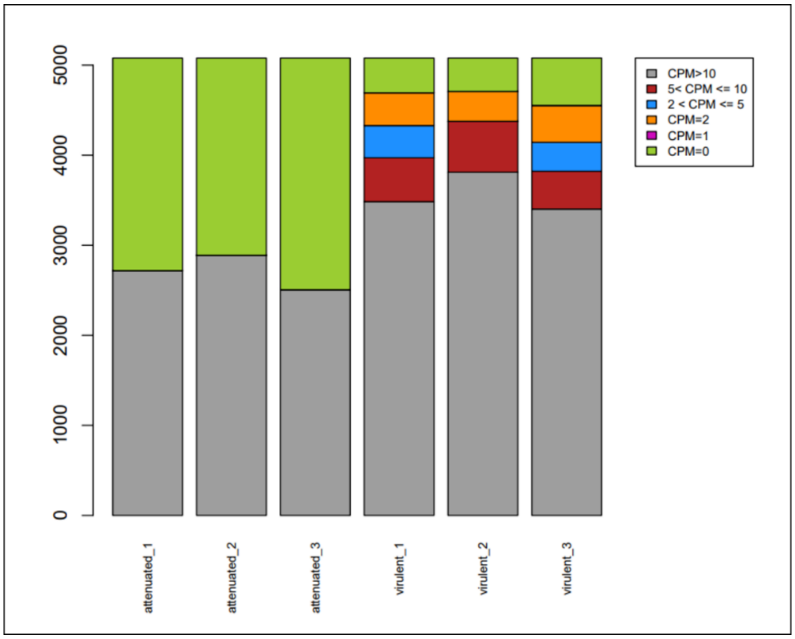

Supplementary Figure S4. CPM Plot. A bar plot for each sample is generated where Counts Per Million for each gene in each biological sample are represented in the attenuated and virulent strains of *B. bigemina*.

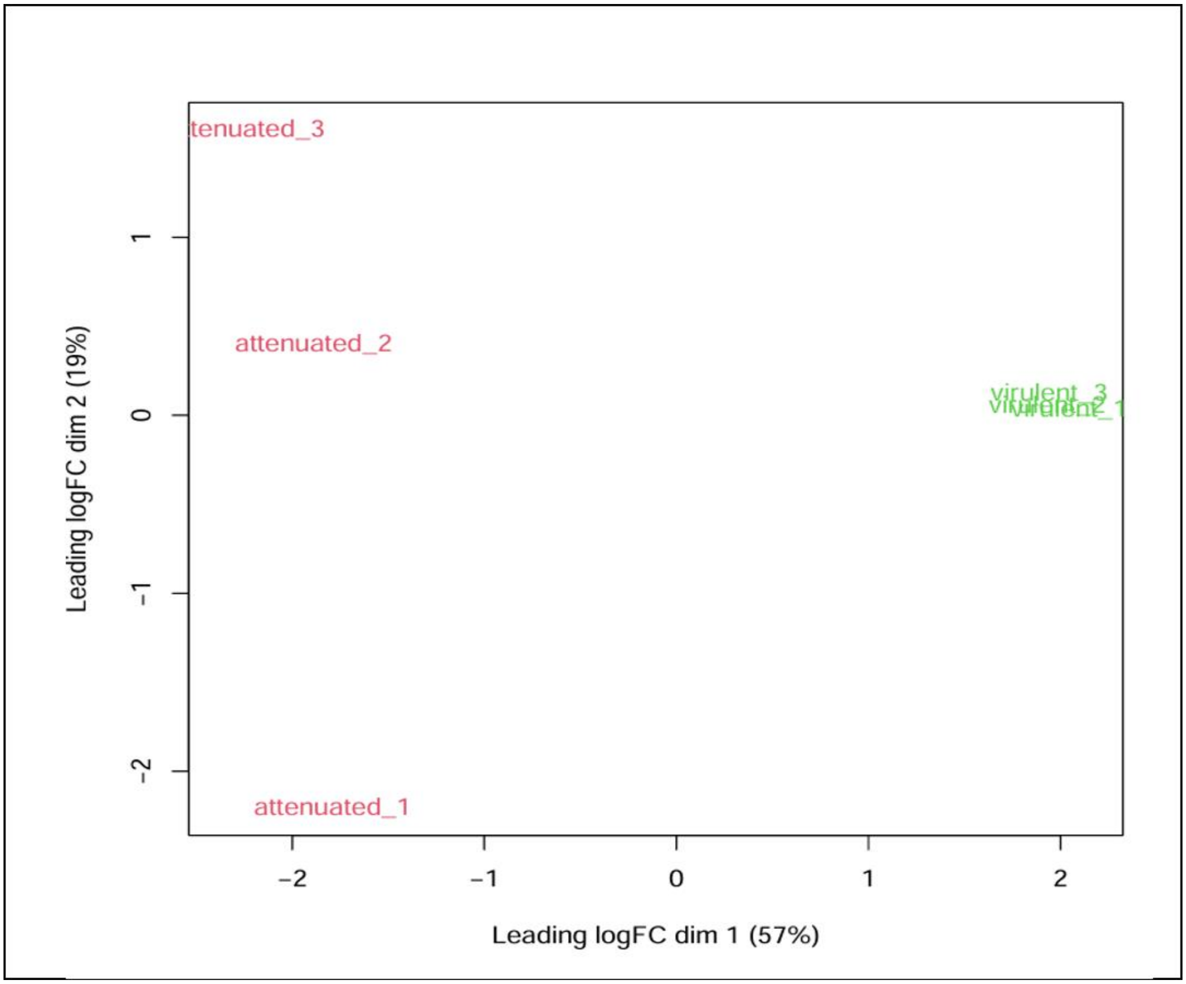

Supplementary Figure S5. Multidimensional scale (MDS) and Normalized TMM counts graphs where the distance between samples and conditions reflect their similarity, for each biological sample of the attenuated and virulent strain of *B. bigemina*.
