# Supplementary material for: Comparative Transcriptome Analysis of Babesia bigemina Attenuated Vaccine and Virulent Strains of Mexican Origin
Source: Vaccines (Basel). 2024 Mar 15;12(3):309. doi: 10.3390/vaccines12030309 (PMC10975891; doi:10.3390/vaccines12030309)
Supplement: Supplementary file 1 [file vaccines-12-00309-s001.zip › Supplementary6_RMSE.pdf]

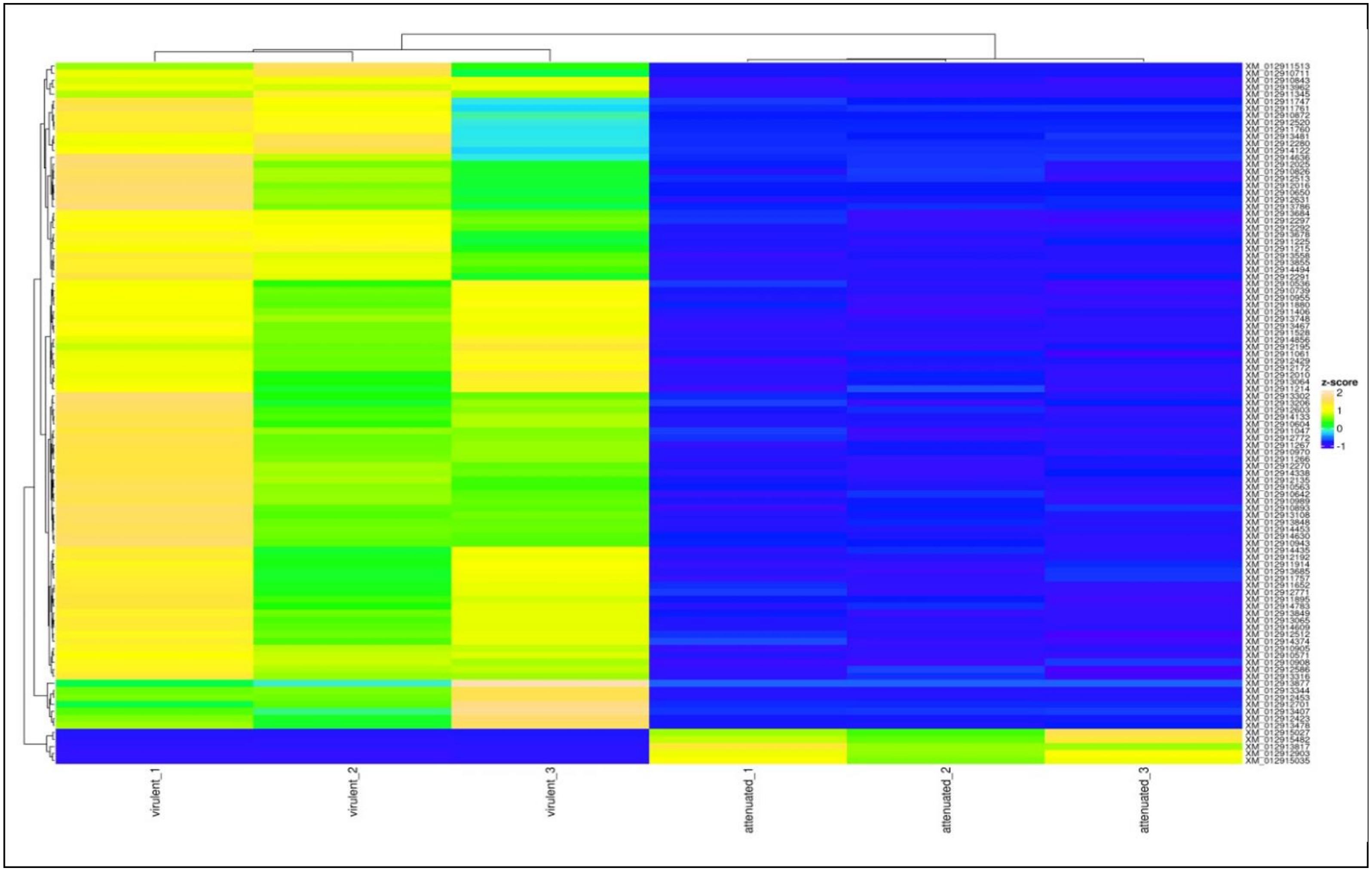

Supplementary Figure S6. Heatmap of the differentially expressed genes in the virulent strain vs. the attenuated strain of *Babesia bigemina*. A higher level of expression can be observed in yellow and a lower expression in blue, with regard to a Z score on the right legend of the map.
