# Supplementary figures and images for: Comparative Transcriptome Analysis of Babesia bigemina Attenuated Vaccine and Virulent Strains of Mexican Origin
Source: Vaccines (Basel). 2024 Mar 15;12(3):309. doi: 10.3390/vaccines12030309 (PMC10975891; doi:10.3390/vaccines12030309)

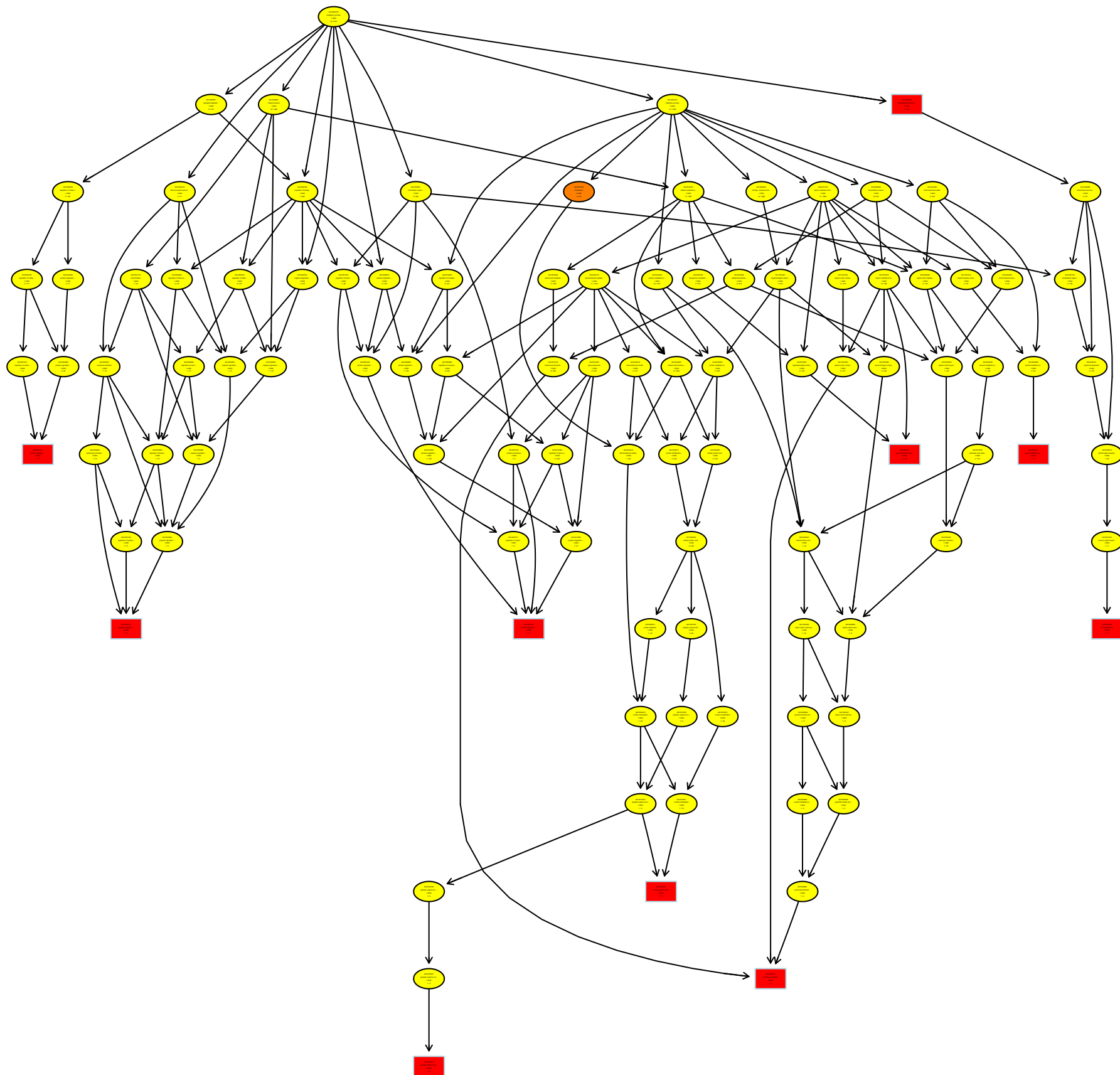

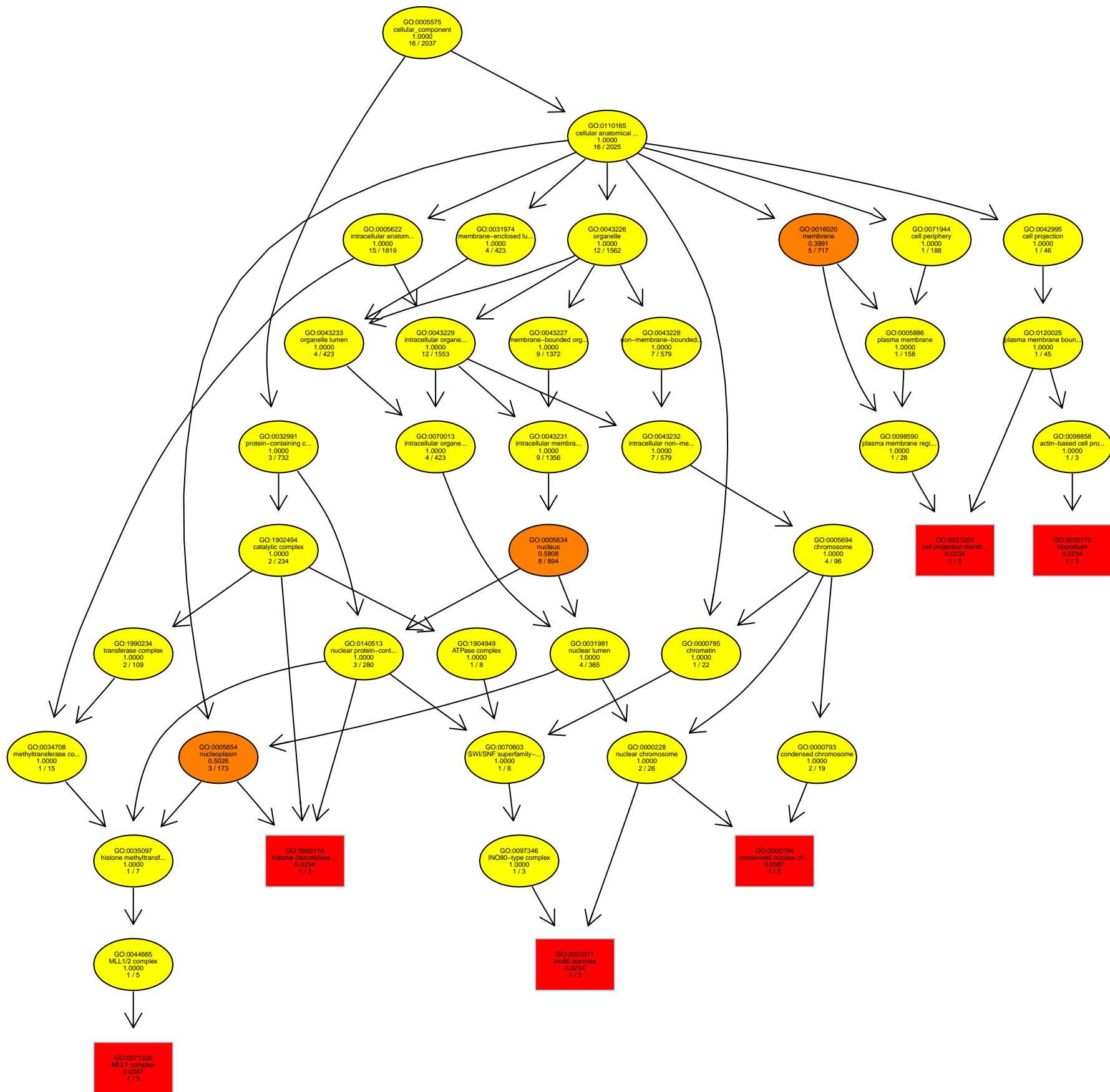

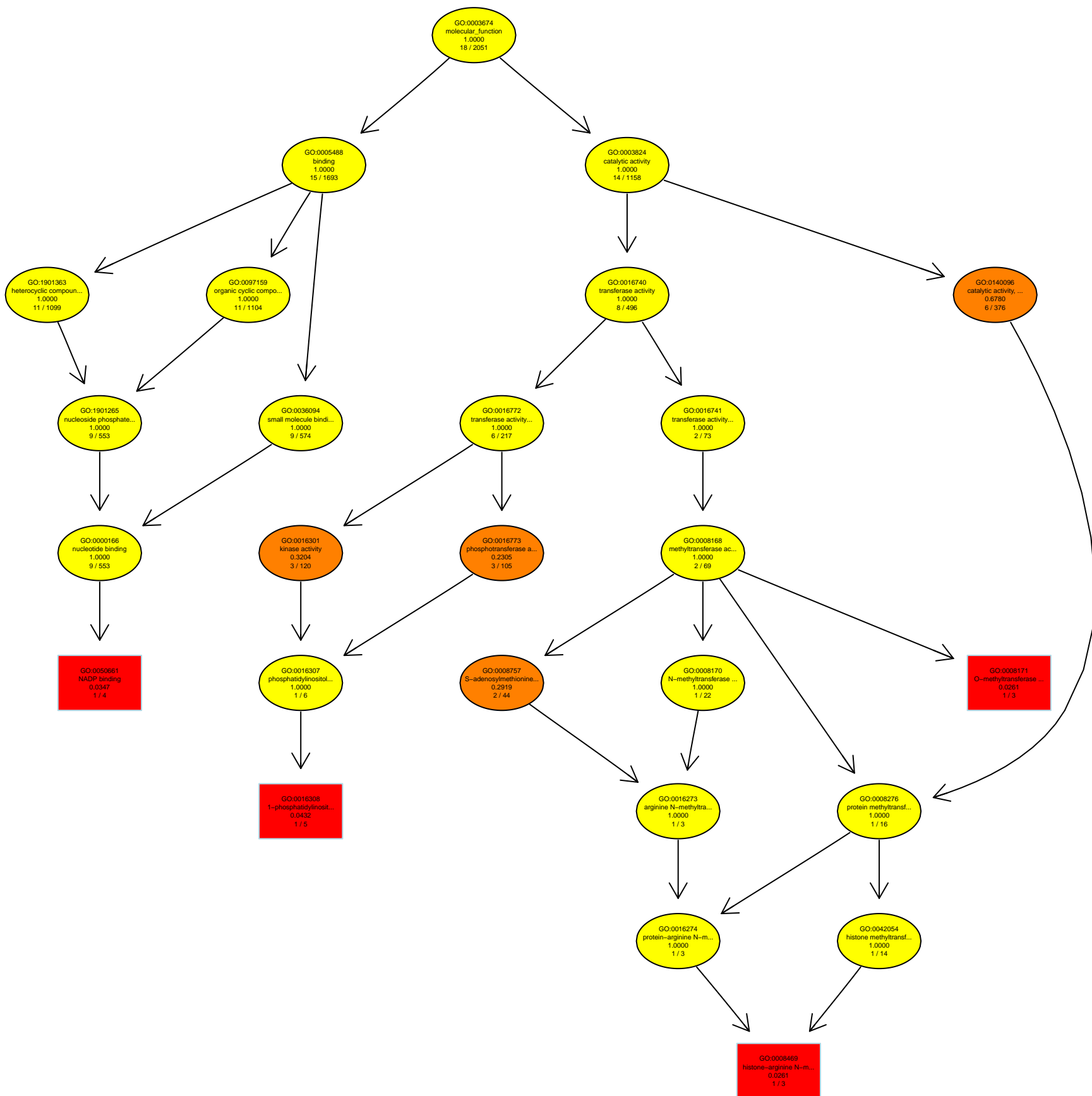

Supplement: Supplementary file 1 [file vaccines-12-00309-s001.zip › Supplementary-RMSE-Figure S7.pdf]
